# Terminal delirium: Is this a diagnosis?

**DOI:** 10.1017/S1478951525100886

**Published:** 2025-10-01

**Authors:** José António Ferraz Gonçalves

**Affiliations:** Departamento de Medicina da Comunidade, Informação e Decisão em Saúde, Faculdade de Medicina, Universidade do Porto, Porto, Portugal

The theme of the article by Uchida et al. (2025), recently published in this journal, is terminal delirium. Although this term has been in use for many years, I am not comfortable with it.

Cognitive failure is common in the last days of life. It is probably normal as part of the dying process, like fatigue or anorexia, reflecting the deterioration of multiple organ systems. In most cases, it is not realistic to try to reverse this decline. However, regarding restlessness or agitation, the situation is different and could not be a natural part of that process. Often, we do not know what is going on, and we call it terminal restlessness, terminal agitation, or terminal delirium as if it were a diagnosis.

Nevertheless, is this a clinical entity? Can it be a diagnosis? Or is it the manifestation of many possible causes? Even if it could be considered a diagnosis, it can only be made a posteriori because only the patient’s death can support that diagnosis. Even then, the diagnosis can be contentious because the cause of the delirium (for example, pneumonia) could also be the cause of death, and letting it run its course can be appropriate or not.

Many years ago, Bruera et al. (1995) carried out a study suggesting that opioid rotation or hydration of patients in their last days of life reduced the frequency of terminal restlessness. They hypothesized that a cause or a cluster of causes, such as dehydration, renal failure, and drug toxicity, might cause delirium at the end of life. Therefore, at least in those cases, terminal delirium could be reversible or preventable and explicable. We will not discuss whether the specific conclusion was right or wrong. The point is that an effort was made to understand the phenomenon.

How many cases are induced simply by poor symptom control or a full bladder? Moreover, how many cases are associated with drug toxicity or drug withdrawal in a phase when swallowing is difficult or impossible, leading to the discontinuation of some drugs? Difficult communication can also occur when the opportunities to express thoughts and feelings or say goodbyes are lost, which can even be aggravated by the medications used to control the agitation. A combination of causes is likely and can occur in many cases.

Therefore, there are many possible causes of terminal delirium, restlessness, or agitation. Nevertheless, what is the problem with this? If, in most cases, it is not appropriate to do anything different, and sedation is the only reasonable way to act, what is the problem?

I am not proposing that we explore the situation in all patients deeply and act up, eventually harming them and inducing more suffering. However, this issue deserves more research because restlessness can cause much suffering to patients, their families, others who watch it as a painful situation, and even staff members. Whether we accept it as a defined clinical entity or a diagnosis, which naturally can occur in the last days of life, we will not recognize the need to research its causes to prevent or reverse it. Therefore, it would be more appropriate to call this situation delirium, restlessness, or agitation in the terminal phase instead of terminal delirium and conduct research to understand it better and so bring more comfort to a substantial part of dying people.
